# The Effects of Sodium Propionate Supplementation in the Diet with High Soybean Meal on Growth Performance, Intestinal Health, and Immune Resistance to Bacterial Infection in Turbot (*Scophthalmus maximus* L.)

**DOI:** 10.1155/2022/8952755

**Published:** 2022-08-31

**Authors:** Huiyuan Sun, Jinjin Zhang, Wentao Wang, Rui Shao, Shufei Liang, Weiqi Xu, Mingzhu Li, Qinghui Ai, Kangsen Mai, Min Wan

**Affiliations:** ^1^Key Laboratory of Aquaculture Nutrition and Feed, Ministry of Agriculture & Key Laboratory of Mariculture, Ministry of Education, College of Fisheries, Ocean University of China, Qingdao, China; ^2^College of Agriculture, Ludong University, Yantai, China; ^3^Pilot National Laboratory of Marine Science and Technology, Qingdao, China

## Abstract

Short-chain fatty acids (SCFAs) are the products of the microbial fermentation of dietary fiber in the intestine. Acetate, propionate, and butyrate are the most abundant SCFA metabolites and play an important role in maintaining host health. This study was aimed at investigating the effects of sodium propionate (NaP) supplementation in the diet with a high proportion of soybean meal (SBM) on the growth, inflammatory status, and anti-infectious ability in juvenile turbot. Four experimental diets were designed: (1) fish meal- (FM-) based diet (control group), (2) SBM protein replacing 45% FM protein in the diet (high SBM group), (3) 0.5% NaP supplementation in the high SBM diet (high SBM+0.5% NaP group), and (4) 1.0% NaP supplementation in the high SBM diet (high SBM+1.0% NaP group). The results confirmed that the fish fed the high SBM diet for 8 weeks showed the decreased growth performance, the typical enteritis symptoms, and the increased mortality responding to *Edwardsiella tarda* (*E. tarda*) infection. However, 0.5% NaP supplementation in the high SBM diet promoted the growth performance of turbot and restored the activities of digestive enzymes in the intestine. Moreover, dietary NaP ameliorated the intestinal morphology, enhanced the expression of intestinal tight junction proteins, improved the antioxidant capacity, and suppressed the inflammatory status in turbot. Finally, the expression of antibacterial components and the resistance to bacterial infection were increased in NaP-fed turbot, especially in high SBM+1.0% NaP group. In conclusion, the supplementation of NaP in high SBM diet promotes the growth and health in turbot and provides a theoretical basis for the development of NaP as a functional additive in fish feed.

## 1. Introduction

The intestine is a complex and dynamic ecosystem, not only for the absorption and metabolism of nutrients but also for the defense against the invading pathogens [1]. The balanced interactions among the epithelial barrier, immune cells, and intestinal microflora are crucial for the maintenance of intestinal health and homeostasis [2]. It has been known that SCFAs are the intestinal microbial fermentation products that are chemically composed of a carboxylic acid moiety and a small hydrocarbon chain [3]. Acetate, propionate, and butyrate are the most abundant SCFA metabolites, among which butyrate is the most widely studied and has been shown to be important in the maintenance of host health [4].

In recent years, propionate has also attracted more attentions. For example, dietary propionic acid enhanced the growth performance, antioxidant ability, and antibacterial effects against *Aeromonas hydrophila* in Nile tilapia (*Oreochromis niloticus*) [5]. Moreover, dietary supplementation of NaP displayed the beneficial effects on mucosal immune responses and the antioxidant defense in zebrafish (*Danio rerio*) [6]. In addition, the mucosal and nonspecific immunity and growth performance of Caspian white fish (*Rutilus frisii kutum*) and common carp (*Cyprinus carpio* L.) were promoted when the fish are fed the NaP-supplemented diet [7, 8].

As the unstable production and continuously rising prices of FM, plant protein sources are widely used in fish feed. SBM has been widely used as a promising alternative protein source in fish feed due to its high protein content, stable supply, and relatively low cost [9]. However, the antinutritional factors contained in SBM, such as protease inhibitors, saponins, and phytic acid, negatively affect the growth performance and intestinal health of fish [10]. It has been known that the impaired epithelial barrier functions, such as increased permeability, reduced gene expression of tight junction proteins, and increased proinflammatory cytokines, appear in the intestine of the fish fed a high proportion of SBM [11]. As an important marine economic carnivorous fish, turbot exhibited obvious growth retardation and enteritis symptoms when fed the high SBM diet [12].

*E. tarda*, a Gram-negative bacterium of the *Enterobacteriaceae* family, has led to severe economic losses in aquaculture [13]. It was reported that the pathological changes after *E. tarda* infection in turbot were extensive [14]. Moreover, zebrafish with intestinal inflammation was more susceptible to *E. tarda*, and more intense acute immune responses were induced in various tissues [15]. Interestingly, our previous study demonstrated that the bactericidal activity of head kidney macrophages (HKMs) in turbot was enhanced when the cells were pretreated with SCFAs, including NaP [16]. Hence, in the present study, different doses of NaP were supplemented into the high SBM diet, and the effects of dietary NaP on the growth, intestinal homeostasis, and antimicrobial ability in turbot were further evaluated.

## 2. Materials and Methods

### 2.1. Animal Ethics

The Laboratory Animal Care Committee in Ocean University of China approved all protocols for animal care and handling procedures in our study. Turbot farming facilities have been optimally configured to ensure refined farming and accommodation, minimizing fish suffering.

### 2.2. Diet Formulation

Four isonitrogenous and isolipidic diets are designed (Table 1). The FM-based diet was named as control, and 45% FM protein was replaced by soybean protein designed as the high SBM diet. In the other two diets, 0.5% or 1.0% NaP (Sigma, St. Louis, MO, USA) was supplemented into the high SBM diet. All the ingredients were grounded into fine powder through a 180 *μ*m mesh and thoroughly mixed with fish oil and soy lecithin, followed by the addition of approximately 30% water to produce stiff dough. Afterwards, the dough was pelleted with an experimental feed mill (South China University of Technology, Guangzhou, China) into 3 mm size of granule feedstuff, dried for about 12 h in a ventilated oven at 45°C, and stored at -20°C for subsequent feeding experiments. The proximate composition of four diets is provided in Table 1.

### 2.3. Fish Maintenance

Juvenile turbot with the initial weight around 15 g were obtained from Longhui Aquatic Product Co. Ltd. in Weihai, Shandong Province, China. The feeding trial was conducted in a flowing water system, and the fish were acclimatized in the experimental system and fed the control diet for 2 weeks. During the acclimation period, fish with malformation, surface damage, and low vitality were excluded. At the beginning of the experiment, the fish with homogenous size were weighed (22.0 ± 0.2 g) and fasted for 24 h before they were randomly allocated to 12 tanks (200 L). Each dietary treatment was randomly assigned to triplicate tanks (40 fish per tank). During the eight-week experiment, the fish were fed twice daily at 7 : 00 and 19 : 00. The water quality was monitored as follows: water temperature 16-18°C, dissolved oxygen > 7.0 mg L^−1^, salinity 27-29‰, ammonia and nitrite < 0.1 mg L^−1^, and pH 7-8. Husbandry and handling of the fish in our study were performed strictly according to the Management Rule of Laboratory Animals (Chinese order no. 676 of the State Council, revised 1 March, 2017).

### 2.4. Sampling

At the end of the feeding experiment, all the fish fasted for 24 h. Turbot from each group were anesthetized and euthanized with 20 mg L^−1^ tricaine. The body weight of fish in each tank were measured before sampling, and four fish from each tank were saved at -80°C for the measurement of body composition. The liver from each fish was collected and weighed for the calculation of the hepatosomatic index. The distal intestine samples of four fish from each tank were collected, followed by fixation with Bouin's fixative solution for further morphological measurement. In addition, the blood samples were collected via the caudal vein from four fish in each tank using heparinized syringes. Furthermore, the serum was obtained by the centrifugation of the blood at 4000*g* for 10 min at 4°C after the blood samples were stored at 4°C overnight. Liver, spleen, and distal intestine samples from four fish per tank were collected and frozen in liquid nitrogen immediately, followed by storage at -80°C for further analysis.

### 2.5. Analysis of Fish Body and Diet Composition

The body composition of turbot was analyzed by using previously described methods [17]. Briefly, the samples were dried at 105°C to determine the moisture contents. Besides, the contents of crude protein and lipids were measured by Kjeltec (TM 8400, FOSS, Sweden) and Soxhlet ether extraction (Buchi 36,680, Switzerland), respectively. The ash contents of the samples were assessed by burning in a muffle furnace for 10 h.

### 2.6. Assay of Enzyme Activities

Total antioxidant capacity (T-AOC), the activity of superoxide dismutase (SOD) and malondialdehyde (MDA) in serum, and the activities of digestive enzymes including trypsin, *α*-amylase, and lipase in the distal intestine were measured by using the commercial kits (Jiancheng, Nanjing, China) according to the manufactures' protocols.

### 2.7. Intestinal Morphology

The fixed tissue segments of fish distal intestine were routinely dehydrated in ethanol, equilibrated in xylene, and embedded in paraffin according to the standard histological procedures. Tissue segments were cut approximately at a thickness of 7 *μ*m by a rotary microtome (Lecia Jung RM 2016, Germany), placed on slides, and stained with hematoxylin and eosin (H&E). The height of villus, the width of lamina propria, and the thickness of muscle layers were determined by analyzing the micrographs with the image analysis software, Image Pro Plus®6.0 (Media Cybernetics, Silver Spring, MD, USA).

### 2.8. Quantitative Real-Time PCR (qRT-PCR) Analysis

Total RNA was extracted by utilizing TRIzol reagent (Accurate Biology, Hunan, China). Furthermore, the quality and quantity of RNA samples were detected by NanoDrop® One spectrophotometer (Thermo Fisher Scientific, USA). The procedure of qRT-PCR was referred to a previous report [18]. The sequences of all primers used in the current study are showed in Table 2.

### 2.9. Bacterial Challenge

At the end of the feeding experiment, twenty-two turbots were randomly selected from different groups. The fish were anesthetized with tricaine (20 mg L^−1^) after being fasted for 24 h, followed by *i.p.* injection with *E. tarda* (1 × 10^7^ CFU in 0.5 mL PBS) according to our previous study [18]. The survival of fish was recorded every 6 h until 36 h.

### 2.10. Sample Collection after Bacterial Challenge

At 12 h post infection (hpi), twelve fish per group were anaesthetized and euthanized with tricaine (20 mg L^−1^), and the spleen and distal intestine were isolated. The spleen was divided into two parts, one of which was used for counting the bacterial burden. Meanwhile, the other parts of spleen and distal intestine were immediately frozen in liquid nitrogen for further analysis.

### 2.11. Bacterial Load (BL) in Spleen

The collected spleen was weighed and homogenized in PBS. The supernatants were plated on LB agar at an applicable dilution. Colony-forming units (CFUs) were counted after bacterial growth for 12 h at 28°C. BL was calculated as CFUs in spleen/the weight of spleen.

### 2.12. HKM Isolation

HKMs were isolated according to the method described by Zhang et al. [16]. In brief, the head kidney of the turbot was isolated and cut into small pieces, followed by passing through a 100 *μ*m nylon mesh. After the obtained cell suspension was centrifuged at 200*g* for 5 min, it was separated on a 34/51% Percoll density gradient by centrifugation at 400*g*. After 30 min, the cells at the interface were collected and dispensed on cell culture plates at 24°C. After 2 h, the nonadherent cells were washed off, and the adherent macrophages were kept in complete medium for further use.

### 2.13. Bacterial Killing Assay

HKMs were incubated with *E. tarda* (cell: bacteria =1 : 1) at 24°C under shaking. After 2 h, the cells were lysed in ice-cold water and vortexed for 90 s. Afterwards, the cell lysates were serially diluted and plated on agar plates overnight at 28°C. In the next day, viable bacteria were counted, and survival rate of bacteria was calculated.

### 2.14. Measurements of Reactive Oxygen Species (ROS), Nitric Oxide (NO), and Lysozyme Activity

The productions of ROS and NO were measured by using commercial kits from Beyotime (Shanghai, China). Lysozyme activity was assessed by a commercial kit from Jiancheng (Nanjing, China). The analysis was performed according to the manufacturer's instructions.

### 2.15. Calculations and Statistical Methods

All data were presented as mean ± SEM. Difference between the means was evaluated by using one-way ANOVA or Tukey's *t*-test. *P* value < 0.05 was considered as statistical significance. All statistical evaluation was performed by using GraphPad Prism 8.0 (GraphPad, San Diego, California, USA).

## 3. Results

### 3.1. Growth Performance and Whole-Body Proximate Composition

As shown in Table 3, no significant difference in the survival rate of turbot among all groups was observed (*P* > 0.05). Compared to the control group, the turbot in the high SBM group displayed a significant decrease in weight gain rate (WGR), special growth rate (SGR), and feed efficiency ratio (FER) (*P* < 0.05), whereas the growth performance of turbot fed the diet containing 0.5% NaP was similar to that in control group. Meanwhile, dietary supplementation of 1.0% NaP in the high SBM diet resulted in a significantly higher SGR (*P* < 0.05), although no difference in WGR and FER was detected. In addition, there was no significant difference in the hepatosomatic index (Table 3) and body composition (Table 4) of turbot among all groups (*P* > 0.05).

### 3.2. Digestive Enzyme Activities in Distal Intestine

**Figure 1:**
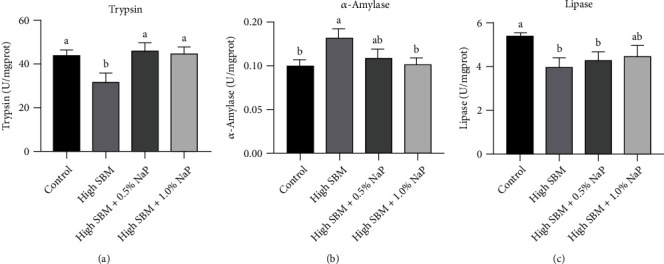
Effects of NaP on the activities of digestive enzymes in distal intestine. (a–c) After the feeding trial, the distal intestine of turbot in different groups was collected, and the activities of trypsin (a), *α*-amylase (b), and lipase (c) in the distal intestine were measured (*n* = 12). Different superscript letters indicated significant difference (*P* < 0.05).

Compared to the control group, the level of trypsin (Figure 1(a)) in distal intestine was significantly decreased in the high SBM group (*P* < 0.05), while the dietary supplementation of 0.5% or 1.0% NaP in the high SBM diet rescued the decline of trypsin activity to a similar level as that in control group. Moreover, a significant upregulation of *α*-amylase activity in distal intestine was detected in the high SBM group (*P* < 0.05), while the dietary supplementation of NaP suppressed the upregulated *α*-amylase activity (Figure 1(b)). Additionally, the lipase activity was significantly restrained in the high SBM group (*P* < 0.05), and NaP seemed no significant effects on the lipase activity (Figure 1(c)).

### 3.3. Intestinal Histomorphology and Mucosal Barrier

**Figure 2:**
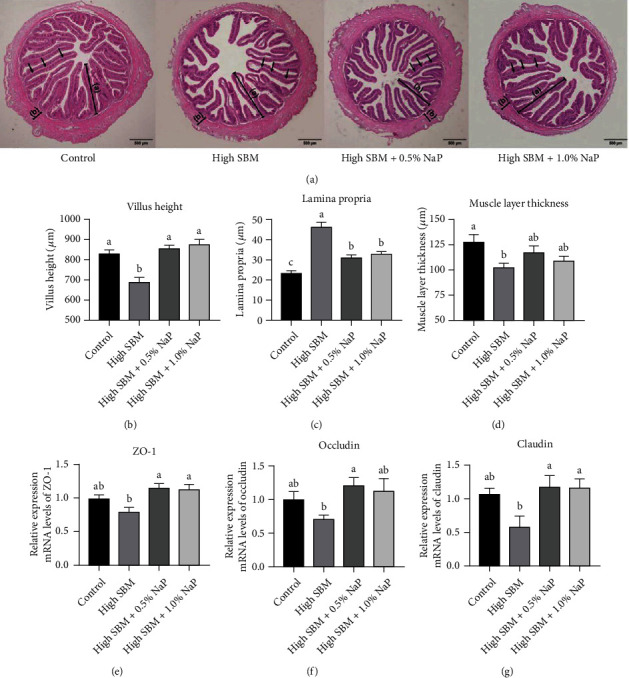
Effects of NaP on intestinal histomorphology and intestinal mucosal barrier. (a) The intestine of turbot in different groups was collected and sectioned. After the fixation by H&E, the morphology of the distal intestines was observed. The images were representative of at least three independent experiments, and scale bar indicates 500 *μ*m; (A) and (B) in the images indicate villus height and muscle layer thickness, respectively; lamina propria is indicated by arrows. (b–d) The micromorphology, including villus height (b), lamina propria (c), and muscle layer thickness (d) of the intestine, was evaluated (*n* = 6). (e–g) The gene expression of ZO-1 (e), occludin (f), and claudin (g) in the intestine was analyzed by qRT-PCR (*n* = 12). Different superscript letters indicated significant difference (*P* < 0.05).

Based on the H&E staining of the distal intestine, intestinal histomorphology was observed among all groups (Figure 2(a)). Further analysis showed that intestinal villus height (Figure 2(b)) and muscle layer thickness (Figure 2(d)) of juvenile turbot in the high SBM group remarkably decreased, whereas intestinal lamina propria increased compared to those in control group (*P* < 0.05) (Figure 2(c)). Similarly, the gene expression of tight junction proteins, including zonula occludens-1 (ZO-1) (Figure 2(e)), occludin (Figure 2(f)), and claudin (Figure 2(g)), was significantly suppressed in the high SBM group. Interestingly, intestinal histomorphology and the gene expression of tight junction proteins in the hindgut of the turbot were reverted to the similar status as control group when 0.5% or 1.0% NaP was supplied in the high SBM diet.

### 3.4. Pro- and Anti-Inflammatory Cytokines

**Figure 3:**
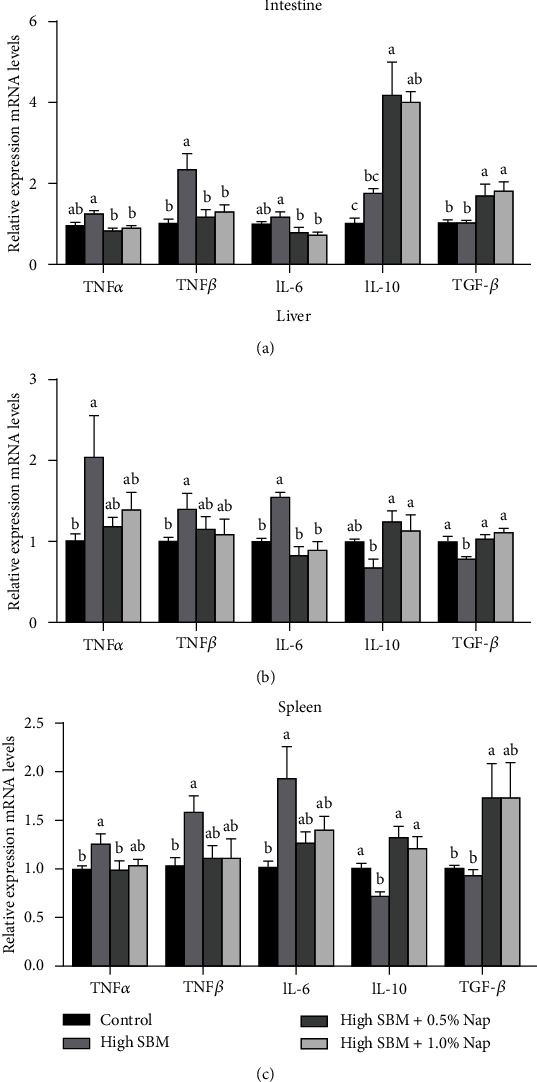
Effects of NaP on the expression of inflammatory cytokines. (a–c) The gene expression of TNF-*α*, IL-1*β*, IL-6, IL-10, TGF*-β* in the distal intestine (a), liver (b), and spleen (c) of turbot in different groups was analyzed by qRT-PCR (*n* = 12). Different superscript letters indicated significant difference (*P* < 0.05).

In order to further examine whether NaP supplementation could alleviate the inflammation induced by high SBM diet, the gene expression of several pro- and anti-inflammatory cytokines in distal intestine, liver, and spleen of turbot was analyzed. As the results shown in Figure 3, the gene expression of proinflammatory cytokines, including tumor necrosis factor-alpha (TNF*-α)*, interleukin-1beta (IL-1*β*), and IL-6 was augmented in the distal intestine, liver, and spleen of turbot in the high SBM group compared to that in control group. However, the genes of the proinflammatory cytokines in juvenile turbot were expressed at similar levels as those in control group when 0.5% or 1.0% NaP was supplied in the high SBM diet.

In contrast, the gene expression of anti-inflammatory cytokines IL-10 and transforming growth factor-beta (TGF-*β)* was significantly restrained in the liver and spleen of the fish in the high SBM group, while the addition of dietary NaP resulted in the significant increase of IL-10 and TGF*-β* expression in the intestine, liver, and spleen of turbot compared to that in the high SBM group (Figure 3).

### 3.5. Antioxidant Enzyme Activities in Serum

**Figure 4:**
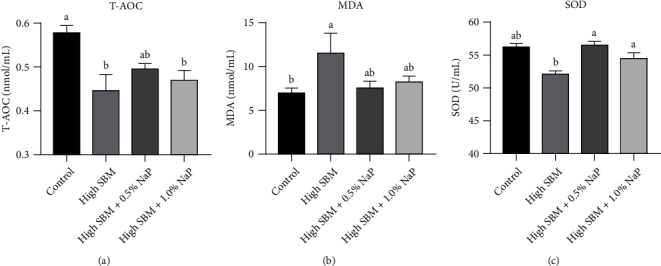
Effects of NaP on antioxidant enzyme activities in serum. (a–c) The serum was obtained as described in Materials and Methods. The levels of T-AOC (a), MDA (b), and SOD (c) of juvenile turbot in different groups were measured (*n* = 9). Different superscript letters indicated significant difference (*P* < 0.05).

Compared to the control group, the levels of T-AOC (Figure 4(a)) and the activity of SOD (Figure 4(c)) in serum were significantly decreased in the high SBM group (*P* < 0.05), while the supplementation of 0.5% NaP in the high SBM diet rescued the decrease of T-AOC and SOD. Moreover, the supplementation of 1.0% NaP in the high SBM diet exhibited a similar effect on SOD activity as high SBM+0.5% NaP group, although no significant effects on T-AOC were detected. Consistently, the dietary supplementation of 0.5% or 1.0% NaP suppressed the elevated MDA in the high SBM group (Figure 4(b)).

### 3.6. The Mortality, Bacterial Loads, and Key Immune Factors after Bacterial Infection

**Figure 5:**
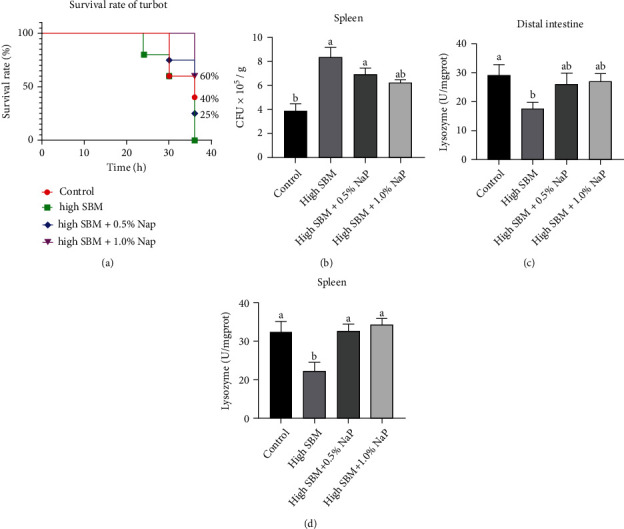
Effects of NaP on the anti-infectious ability in turbot after bacterial infection. After the feeding trial, a part of turbot were infected by *i.p*. injection with *E. tarda* (1 × 10^7^ CFU/fish). (a) The mortality of the turbot was recorded every 6 hours (*n* = 10). (b) The viable bacteria in the spleen at 12 hpi were counted (*n* = 12). (c and d) The lysozyme activities in the distal intestine (c) and spleen (d) of infected turbot were measured (*n* = 12). Different superscript letters indicated significant difference (*P* < 0.05).

To further analyze the effects of dietary NaP supplementation on the anti-infectious ability of juvenile turbot, the fish in different groups were intraperitoneally injected with *E. tarda*, and the mortality of the fish was recorded. As shown in Figure 5(a), all fish in the high SBM group died at 36 hpi, while the survival rate of the fish in control group was around 40%. Interestingly, around 60% fish in the high SBM+1.0% NaP group survived at 36 hpi, although the fish in the high SBM+0.5% NaP group exhibited a higher mortality than control group. Moreover, significantly more *E. tarda* in the spleen of infected turbot in the high SBM group at 12 hpi was detected compared to that in control group. Nonetheless, the bacterial load was repressed in the spleen of the fish with dietary NaP supplementation (Figure 5(b)). Additionally, the lysozyme activity in the infected fish of the high SBM group was lowest, and dietary NaP enhanced the lysozyme activity to the similar level as control group (Figure 5).

### 3.7. The Bactericidal Activity and the Antibacterial Effectors in HKMs

**Figure 6:**
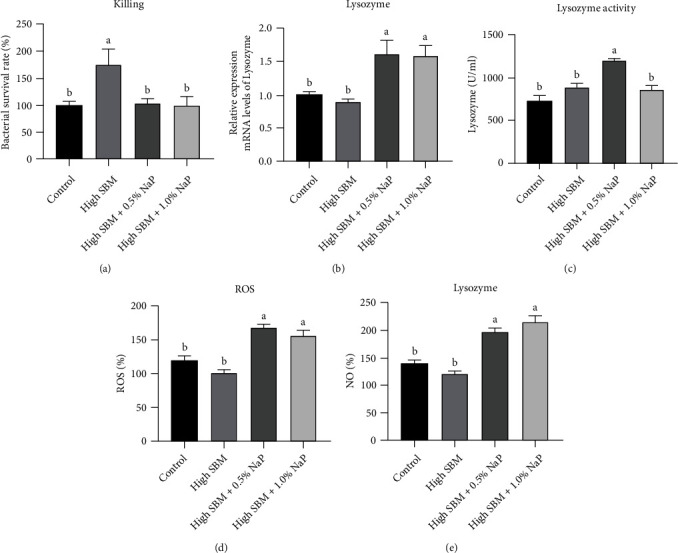
Effects of NaP on the bactericidal activity and the production of antibacterial effectors in HKMs. After the feeding trial, HKMs were isolated and cultured for 24 h. (a) *E. tarda* was added (cells: bacteria =1 : 1) and coincubated for 2 h. The bactericidal activity of HKMs was assessed (*n* = 6). (b and c) The gene expression of lysozyme (b) and enzyme activity (c) in the cells were measured (*n* = 6). (d and e) *E. tarda* was added (cells: bacteria =1 : 1) and coincubated for 2 h; the production of ROS (d) and NO (e) in the cells was analyzed (*n* = 6). Different superscript letters indicated significant difference (*P* < 0.05).

Finally, HKMs were isolated from juvenile turbot after the feeding trail and coincubated with *E. tarda* for 2 h. As shown in Figure 6(a), the bacterial load in HKMs from the fish in the high SBM group was significantly higher than that in control group (*P* < 0.05), while the bacterial loads in HKMs from the fish in the groups supplemented with 0.5% or 1.0% NaP were significantly lower than that in the high SBM group and similar to that in control group. Furthermore, the gene expression of lysozyme (Figure 6(b)) and lysozyme enzyme activity (Figure 6(c)) in the 0.5% NaP group and 1.0% NaP group were significantly higher than that in control group (*P* < 0.05). In addition, the production of *E. tarda*-induced ROS (Figure 6(d)) and NO (Figure 6(e)) in HKMs from the 0.5% NaP group and 1.0% NaP group was significantly higher than the control group (*P* < 0.05).

## 4. Discussion

The current study has demonstrated that the supplementation of NaP in high SBM diet significantly increases the growth performance, antioxidant ability, and intestinal barrier function in turbot. Meanwhile, propionate alleviates the inflammatory symptoms and promotes the anti-infective ability in juvenile turbot. Our results have highlighted the potentials to use propionate as a feed additive to reduce foodborne enteritis and promote the growth and immunity in marine fish.

It has been reported that the diet with a high proportion of SBM can induce poor growth performance in many fish, including turbot [12, 19–21]. Our study confirmed that the growth of the turbot in the high SBM group was significantly suppressed. In contrast, the growth performance of NaP-supplemented groups, especially the 0.5% NaP group, recovered to the level even higher than that in the control group, which proved the beneficial effects of propionate on promoting the growth of turbot (Table 3). Interestingly, we identified that the activities of digestive enzymes, such as trypsin, *α*-amylase, and lipase, were significantly interfered in the intestine of the fish fed the high SBM diet (Figure 1). Trypsin has been known as a key enzyme for feed utilization and growth in fish due to its role in the protein digestion processes [22]. According to the previous reports, the antinutritional factors in SBM, such as protease inhibitors and phytic acid, could inactivate trypsin [23, 24]. Consistently, the trypsin activity in the intestine of the turbot fed the high SBM diet was significantly repressed. However, the trypsin activity was recovered to the similar level as that in the control group when 0.5-1.0% NaP was supplemented in the high SBM diet, which could partly explain the beneficial effects of NaP on the growth of turbot. In contrast to trypsin, the activity of amylase in high SBM group was increased. A similar result was obtained in the previous study showing that the amylase activity in the digesta of Atlantic cod (*Cadus morhua*) fed the SBM diet was higher than that in the FM group, caused by the less wheat in the SBM diet than that in the FM diet [25]. Different from trypsin and amylase, dietary NaP supplementation exhibited no significant influence on SBM-suppressed activity of lipase in the intestine, which is consistent to the previous study on the silver catfish (*Rhamdia quelen*), showing that lipase activity was unaffected by dietary NaP [26].

Our study has showed that 0.5% or 1.0% NaP supplementation in the high SBM diet reduces the damage of intestinal mucosal barrier caused by the high SBM diet (Figure 2). The damage of intestinal mucosal barrier could result in the increase of intestinal permeability, so that pathogenic bacteria, antigenic substances, and toxins in the intestine are more likely to invade the body [27]. It has been known that intestinal mucosal barrier is mainly composed of epithelial cells and tight junctions between cells, which are composed of a variety of tight junction proteins, such as occludin, claudins, and ZO-1 [27, 28]. The studies on mammals showed that NaP increased the expression of tight junction proteins claudin-5, occludin, and ZO-1 in human brain microvascular endothelial cells [29]. NaP also elevated the expression of occludin and claudin-3 to reduce the blood-milk barrier permeability via NF-*κ*B signaling pathway and the inhibition of histone deacetylase [30]. In addition, NaP stimulated the spreading and polarization of intestinal epithelial cells, leading to an increase in cell speed and persistence to promote epithelial renewal and repair [31]. All these results have suggested the potentials that propionate promotes the intestinal health.

Notably, the expression of the proinflammatory cytokines in the fish fed the high SBM diet was significantly enhanced not only in the intestine but also in the liver and spleen (Figure 3), indicating that the systemic inflammation was induced. Intriguingly, the addition of 0.5-1.0% NaP in the high SBM diet downregulated proinflammatory cytokines and upregulated the expression of anti-inflammatory cytokines in different organs of the turbot. Recent studies have indicated that gut microbiota-derived SCFAs can exert their influence in peripheral tissues. The blood concentrations of the three SCFAs in higher animals ranged from 60 to 440 *μ*mol/L [32, 33]. Although the level of SCFAs in the peripheral circulation was low, it still played an important physiological role as a signaling molecule [34]. For example, Trompette et al. demonstrated that the levels of SCFAs in the blood of the mice fed a high-fiber diet increased, which in turn inhibited allergic inflammation in their lungs [35]. In addition, the enhanced concentration of propionic acid in the hepatic portal vein could effectively reduce the inflammatory response in the liver [36]. Currently, little has been known about the extraintestinal action and regulatory mechanism of SCFAs in fish.

Furthermore, dietary NaP supplementation seemed to restore the antioxidant capacity of the fish to a certain extent (Figure 4). In previous studies, oral administration of NaP could ameliorate dextran sulfate sodium- (DSS-) induced colitis of mice mainly by improving intestinal barrier functions, reducing inflammation and oxidative stress [37]. In addition, NaP inhibited lipopolysaccharide- (LPS-) induced oxidative stress and inflammatory responses by lowering the expression of cyclooxygenase-2 (COX-2) and inducible nitric oxide synthase (iNOS). Meanwhile, NaP increased the production of antioxidant enzymes, such as manganese superoxide dismutase (MnSOD) and heme oxygenase-1 (HO-1) to reduce oxidative damage caused by H_2_O_2_ stimulation [38]. Interestingly, a recent study in zebrafish elucidated that dietary NaP supplementation in a high-fat diet might cause intestinal damage in zebrafish. Further analysis found that propionate crossed the mitochondrial intima and acted as a precursor to propionyl-COA generation. In the case of high fat, the accumulation of propioyl-CoA appeared to enhance the propionylation of SOD, thus reducing the activity of antioxidant enzymes and leading to intestinal oxidative damage [39].

In an inflammatory state, the intestinal homeostasis in fish is interrupted, and the fish is more susceptible to the attack of pathogenic microorganisms, and pathogen could induce more intense and acute immune responses in various tissues [15]. Our study has demonstrated that dietary NaP supplementation in the high SBM diet increases the survival rate of the infected turbot and lowers the bacterial loads in the spleen (Figure 5). Consistently, previous studies showed that dietary propionic acid improved the resistance of *Oreochromis niloticus* to *Aeromonas hydrophila* [5]. Moreover, zebrafish fed NaP-supplemented diets significantly reduced the mortality of *Aeromonas veronii* infection [39]. It has been known that macrophages in teleost fish can rapidly kill pathogens through phagocytosis and the production of antimicrobial factors, such as lysozyme, ROS, and NO [40]. Recently, our research team elucidated that the bactericidal activity of HKMs in turbot was significantly enhanced *in vitro* after the incubation with SCFAs including NaP [16]. The current study has further confirmed that propionate improves the expression of the antimicrobial components, such as lysozyme, ROS, and NO in HKMs, and promotes the bactericidal ability of HKMs ex vivo (Figure 6). The study on chicken macrophages has proved that three SCFAs, including butyrate, propionate, and acetate, exhibit a strong synergistic effect on the enhancement of the expression of antimicrobial peptide, thus improving the ability of the host to fight against infection [41]. Considering that there are a large number of macrophages in the intestinal tract and other peripheral tissues, we believe that the regulation of the bactericidal ability in macrophages by NaP plays a very important role in promoting the anti-infection ability of fish. However, the contents of lysozyme, ROS, and NO in the HKMs of turbot in high SBM group was almost same to those in control group, suggesting that other antimicrobial factors or pathways in the HKMs of turbot in high SBM group could be disturbed.

It is noteworthy that propionate has been widely used as an important preservative in food [42, 43]. In animal husbandry, propionate was used as a feed mineral supplement to inhibit mycotoxin production and prevent milk fever in dairy cows [44]. In aquaculture, calcium propionate has been commonly used as a fungicide in feed to inhibit the growth of molds, and the dosage is usually 0.1% [45, 46]. In this study, 0.5% or 1.0% NaP was further supplemented on the basis of 0.1% calcium propionate addition in the diet. According to our results, the survival and body composition of turbot during the feeding period were not significantly affected under two concentrations of NaP in the diet (Table 4). Nonetheless, 0.5% NaP supplementation in high SBM diet significantly contributed to promote growth performance and improve intestinal health in turbot, while 1.0% NaP supplementation was effective to alleviate enteritis and enhance anti-infectious ability in turbot fed high SBM diet.

## 5. Conclusion

Our study has elucidated that the supplementation with 0.5% and 1.0% NaP in a high SBM diet contributes to intestinal health in turbot. Moreover, 0.5% NaP supplementation in high SBM diet is recommended for growth promotion in turbot, while 1.0% NaP supplementation in high SBM diet is beneficial for resistance to bacterial infection in turbot. The current study has provided a theoretical basis for the development of NaP as a functional feed additive for marine fish to treat foodborne enteritis and promote the growth and immunity in fish.

## Figures and Tables

**Table 1:** Formulation and proximate composition of the experimental diets (air dry basis, g/kg).

Ingredient	Control	High SBM	High SBM+0.5% NaP	High SBM+1.0% NaP
Fish meal^a^	600.0	330.0	330.0	330.0
Soybean meal^b^	0.0	356.9	356.9	356.9
Wheat gluten meal^c^	44.6	87.5	87.5	87.5
Wheat meal^d^	225.9	41.9	41.9	41.9
Beer yeast^e^	20.0	20.0	20.0	20.0
Fish oil	30.5	53.1	53.1	53.1
Soy lecithin	25.0	25.0	25.0	25.0
Monocalcium phosphate	5.0	5.0	5.0	5.0
Vitamin premix^f^	15.0	15.0	15.0	15.0
Mineral premix^g^	15.0	15.0	15.0	15.0
Choline chloride (99%)	2.5	2.5	2.5	2.5
Calcium propionate	1.0	1.0	1.0	1.0
Ethoxyquin	0.5	0.5	0.5	0.5
Attractants^h^	10.0	10.0	10.0	10.0
Sodium alginate	5.0	5.0	5.0	5.0
Microcrystalline cellulose	0.0	31.6	26.6	21.6
Sodium propionate^i^	0.0	0.0	5.0	10.0
*Proximate composition (dry matter basis, g/kg)*				
Crude protein	517.1	512.2	507.6	503.8
Crude lipid	119.2	118.1	123.1	126.2
Crude ash	119.4	103.7	108.6	110.7

Abbreviations: SBM: soybean meal diet; NaP: sodium propionate. ^a^Purchased from Qingdao Seven Great Bio-tech Company Limited (Qingdao, China), crude protein: 711.6 g/kg, crude lipid: 103.4 g/kg (dry matter basis). ^b^Purchased from Qingdao Seven Great Bio-tech Company Limited (Qingdao, China), crude protein: 538.4 g/kg, crude lipid: 27.2 g/kg (dry matter basis). ^c^Purchased from Qingdao Fulin Company Limited (Qingdao, China), crude protein:858.9 g/kg, crude lipid: 22.5 g/kg (dry matter basis). ^d^Purchased from Qingdao Fulin Company Limited (Qingdao, China), crude protein:199.9 g/kg, crude lipid: 29.0 g/kg (dry matter basis). ^e^Purchased from Qingdao Fulin Company Limited (Qingdao, China), crude protein:477.6 g/kg, crude lipid: 17.2 g/kg (dry matter basis). ^f^Vitamin premix (mg kg^−1^ diet): retinyl acetate (500000 IU/g), 32; thiamine HCl (98%), 25; riboflavin (80%), 45; niacin (99%), 200; D-calcium pantothenate (98%), 60; pyridoxine HCl (99%), 20; inositol (98%), 800; folic acid (98%), 20; cyanocobalamin (1%), 10; ascorbic acid (35%), 120; cholecalciferol (500000 IU/g), 5; *ɑ*-tocopheryl acetate (50%), 240; biotin (2%), 60; menadione sodium bisulphite (51%), 10; ethoxyquin (100%), 3; microcrystalline cellulose (100%), 11470. ^g^Mineral premix (mg kg^−1^ diet): CoCl_2_·6H_2_O (1%), 50; CuSO_4_·5H_2_O (25%), 10; FeSO_4_·H_2_O (30%), 80; ZnSO_4_·H_2_O (34.50%), 50; MnSO_4_·H_2_O (31.80%), 45; MgSO_4_·H_2_O (15%), 1200; Na_2_SeO_3_ (1%), 20; calcium iodine (1%), 60; zeolite powder, 13512. ^h^Betaine: DMPT: threonine: glycine: inosine-5-diphosphate trisodium salt =4: 2: 2: 1: 1. ^i^Purchased from Sigma-Aldrich Co. (USA). The batch number was 303,410–500G, and the purity was more than 98%.

**Table 2:** Primer sequences used for qRT-PCR.

Gene	Forward primers (5′–3′)	Reverse primers (5′–3′)
Occludin	CGTGCGTTGCCTCCACTCTC	CTCCCACTCCGCCCATCTGC
ZO-1	CCCAAGAGGAGAAGAAGTAA	TCAAAATGTGTCCGAATGTA
Claudin	GCCAGATGCAGTGTAAGGTC	CCGTCCAGGAGACAGGGAT
IL-1*β*	GGCAGACCCCTTGAAGAATA	TGGTGAACCCTTCCCATTAT
TNF-*α*	GGGTGGATGTGGAAGGTGAT	GGCCTCTGTTTGGCTTGACT
IL-6	TTCTTTTATCCCAACCCCGC	TTCTGGTCCCGCTTCGTTTC
IL-10	CCACGCCATGAACAGCATCCT	ACATCGGACTTGAGCTCGTCGAA
TGF-*β*	CTGCAGGACTGGCTCAAAGG	CATGGTCAGGATGTATGGTGGT
Lysozyme	GAGACTGGAACCCACACAGGAACG	CTGCTCTCCGCTCCAATCAGGAA
*β*-Actin	GCGTGACATCAAGGAGAAGC	TGGAAGGTGGACAGGGAAGC

**Table 3:** Effects of dietary NaP on the growth performance of juvenile turbot.

Diet	Control	High SBM	High SBM+0.5% NaP	High SBM+1.0% NaP
Survival rate (%)	97.50 ± 1.44	93.33 ± 0.83	95.83 ± 0.83	92.50 ± 3.82
Initial body weight (g)	21.90 ± 0.12	22.11 ± 0.25	22.12 ± 0.04	21.95 ± 0.04
Final body weight (g)	76.57 ± 2.08^a^	65.78 ± 3.57^b^	77.55 ± 2.59^a^	71.33 ± 2.38^ab^
Weight gain rate (%)	247.00 ± 12.12^a^	199.20 ± 11.40^b^	250.00 ± 8.70^a^	225.00 ± 11.95^ab^
Special growth rate (%/d)	1.85 ± 0.04^a^	1.59 ± 0.04^b^	1.90 ± 0.04^a^	1.80 ± 0.06^a^
Feed efficiency ratio	1.15 ± 0.03^a^	1.05 ± 0.02^b^	1.18 ± 0.03^a^	1.05 ± 0.03^b^
Hepatosomatic index (%)	1.34 ± 0.07	1.33 ± 0.06	1.25 ± 0.05	1.40 ± 0.03

Values were mean ± SEM (*n* = 3), and values within the same row with different letters were significantly different (*P* < 0.05). Survival rate, SR (%) = 100 × (the final number of fish/the initial number of fish). Weight gain rate, WGR (%) = 100 × [(final body weight (g) − initial body weight (g))/initial body weight (g)]. Specific growth rate, SGR (%/d) = 100 × (ln final body weight (g)–ln initial body weight (g))/days. Feed efficiency ratio, FER = weight gain (g)/total amount of feed consumption (g). Hepatosomatic index, HIS (%) = 100 × liver weight (g) of final individual fish/final individual weight (g).

**Table 4:** Effects of dietary NaP on the body composition of turbot (fresh weight, g/kg).

Diet	Control	High SBM	High SBM+0.5% NaP	High SBM+ 1% NaP	*P* values
Moisture	774.2 ± 7.2	784.2 ± 4.2	779.8 ± 4.5	778.3 ± 2.8	NS
Crude lipid	34.5 ± 1.3	34.6 ± 2.8	37.5 ± 1.3	36.4 ± 2.8	NS
Crude protein	142.5 ± 3.8	143.2 ± 2.7	142.8 ± 4.1	140.3 ± 2.0	NS
Crude ash	37.9 ± 1.3	38.0 ± 0.7	38.7 ± 0.8	39.2 ± 0.7	NS

Values were mean ± SEM (*n* = 3). NS: no significance (*P* > 0.05).

## Data Availability

The data used to support the findings of this study are included within the article.
